# Dejun Yu: a patriotic botanist and his contributions

**DOI:** 10.1007/s13238-017-0381-y

**Published:** 2017-02-21

**Authors:** Hong Jiang

**Affiliations:** Center for Culture, Science and Technology, Sichuan University, Chengdu, 610065 China

**Figure 1:**
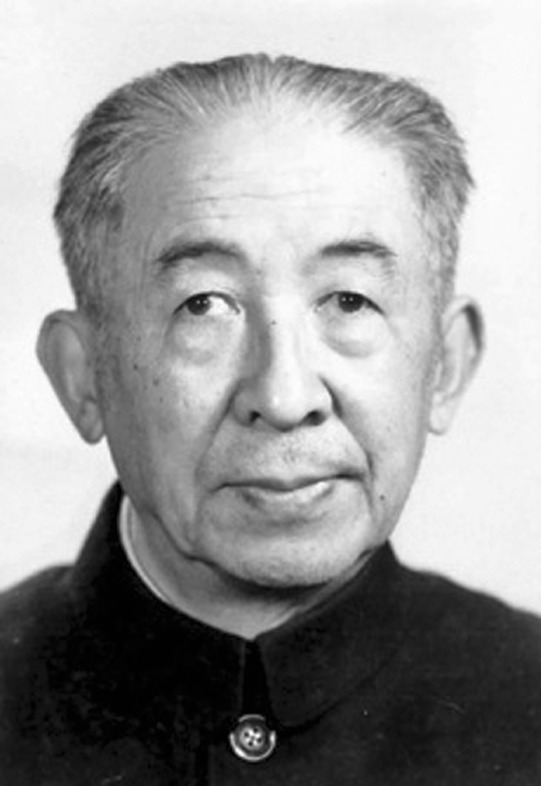
Dejun Yu (Te-tsun Yu, Feb. 1, 1908–July 14, 1986)

Dejun Yu (Te-tsun Yu, Feb. 1, 1908–July 14, 1986) was a famous plant taxonomist and horticulturist in China as well as an expert on botanical gardens (Fig. 1). As a patriotic scientist, Dejun Yu refused a position in Britain and returned to China without hesitation in 1950. He devoted his whole life to the botanical sciences. His contributions went far beyond advancing botany, however, extending to construction of botanical gardens and the exploration of plant resources, especially fruit trees in China. His research was closely connected to the scientific and economic development of his country.

Dejun Yu began his career in plant taxonomy at the Fan Memorial Institute of Biology upon his graduation from the Department of Biology at National Beiping Normal University in 1931. His first important collecting task was three-year plant hunting in western Sichuan province from 1932 to 1934. He started his second hunting journey with his team to northwest Yunnan province, which went from 1937 to 1939, three years later. The regions he went to in the 1930s suffered from warlord conflicts, serious poverty, isolation, poor transportation and complicated natural environment, but also had high biodiversity. Such explorations, therefore, meant a lot of dangers and challenges on one hand, and great contributions to the progress of plant taxonomy and investigation in China on the other hand. Dejun Yu often hired local people as carriers, guides and translators, and got along well with them. A villager from the Dulong River valley, Zhiqing Kong, once shared their stories. Dejun Yu was the first botanist collecting plants around Dulong River region. Sometimes he even risked his life to collect species. He also always worked hard on recording and organizing specimens after long trudges in the jungle. He established deep friendship with local people including Zhiqing Kong, teaching him mandarin and helping him enter school, which made Kong become the first educated Dulong person (Kong & Zhou, 1986).

Dejun Yu was both a field botanist and an armchair botanist, and often shifted between the two when he focused on his taxonomic research, including families of Rosaceae, Leguminosae, Begoniaceae and Theaceae. He and his students spent over 20 years on the taxonomy of Rosaceae plants, organizing and checking nearly 300,000 specimens collected all over the country. However, this did not mean they completely relied on specimens. They often travelled to investigate plants *in situ*, especially to identify new species. In 1980, Dejun Yu and his student Chaoluan Li found a new genus named *Taihangbia* by identifying a specimen. They affirmed it by travelling to Taihang Mountain and finding its type species *Taihangbia rupestris*, and then published this genus. His team finally compiled three volumes of *Flora Republicae Popularis Sinicae* (FRPS) on this family, Vol. 36–38 (Anonymous, 1986). Supported by the Kew in London, UK, Dejun Yu did research and received advanced training in taxonomy of higher plants, horticulture, establishment and management of botanical gardens at the Royal Botanic Garden Edinburgh and the Kew from 1947 to 1950. In Britain, he was inducted into International Camellia Society because of his highly praised paper *Camellia plants and their cultivars in Yunnan Province*, *China*. From 1978 to 1986, he served as the acting editor-in-chief and then editor-in-chief of FRPS, as many as 35 volumes of which were published during that period. His another contribution to taxonomy was his research on fruit trees. In 1979 he compiled *Taxonomy of Chinese Fruit Trees*, collecting 59 families and 670 species, including detailed information on morphology, chromosome number, phenology, distribution, and cultivars. This monograph also won him the first prize of “National Excellent Monographies of Science & Technology” in 1982.

**Figure 2:**
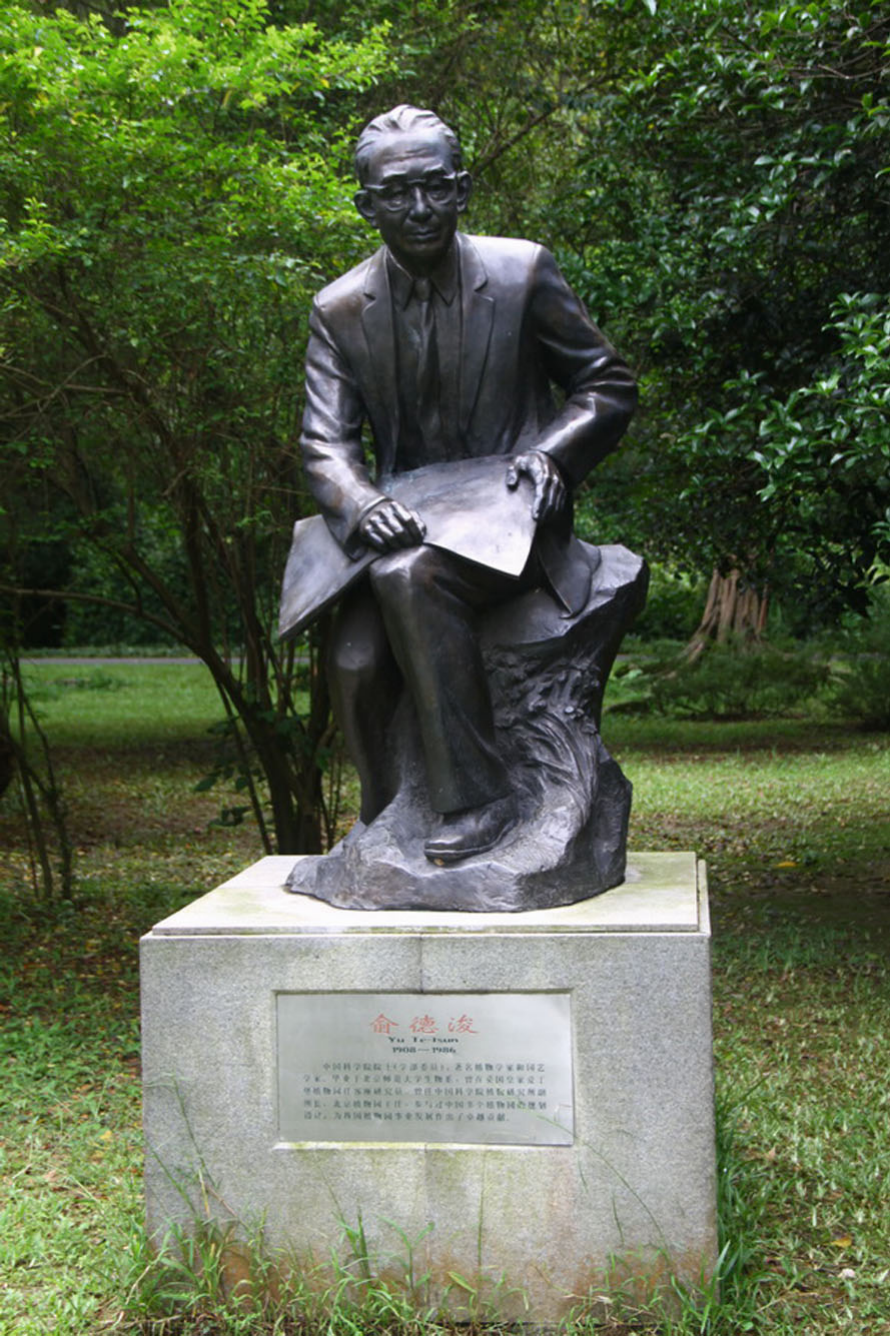
Bronze Statue of Prof. Dejun Yu located in South China Botanical Garden, Chinese Academy of Sciences (SCBG, CAS)

During his visit in Britain, Dejun Yu realized how important role botanical gardens played in research, economic development and science popularization. He devoted himself to the foundation of the Beijing Botanical Garden (BG) as soon as he returned to China. In order to find a proper place for the new garden, Dejun Yu led a team of experts to explore a dozen places all over Beijing city and compare them, and finally built it at the foot of Xiangshan Mountain. He was then appointed as the director of Beijing BG, engaged in the design, planting, building and daily management of it. Besides founding Beijing BG, he also greatly helped with the establishment of quite a few botanical gardens in China including: Lushan BG, Wuhan BG, Xishuangbanna Tropical BG, Hangzhou BG, etc. (Fig. 2). Along with the development of botanical gardens in China, he compiled *Handbook of Botanical Gardens*, an important guide to the building of modern botanical gardens in China, started the journal *Collections of Plant Introduction and Domestication*, to introduce research and progress achieved by these newly built gardens, and published the album of painting *Chinese Botanical Gardens*. As far as the introduction of plants is concerned, Dejun Yu proposed six principles of recording for each plant: 1. Introducing year, number and origin; 2. Accurate Chinese and Latin names; 3. Picture of planting and ID card; 4. Complete phenological records; 5. Detailed biological description; 6. Photo, seed samples and exsiccate (Anonymous, 1986). These requirements were identified with his ideas on the aims of botanical gardens, especially those under the administration of Chinese Academy of Sciences, namely research first, followed by protection of plants species and especially cultivation of economic plants for the need of agriculture.

Besides the taxonomy and botanical gardens, Dejun Yu’s interests in botany also extended to plant resources, especially research and cultivation of fruit trees and ornamental plants, both closely related to his taxonomic research. During Dejun Yu’s life, China experienced wars, deep poverty and the “Great Cultural Revolution”. He was very sensitive to species that may be cultivated as economic or ornamental plants. Many species of Rosaceae and Theaceae, the two families he specialized in, have been cultivated in agriculture and horticulture. According to his research, Chinese plants had been greatly introduced around the world. There were over two hundred fruit species around the world native to China. Various Chinese flower plants were blooming in European gardens, which won China a name of “Mother of Gardens” (Yu, 1985). Specifically, he contributed to the exploration of plant resource by means of collecting wild species with potential for cultivation during his plant hunting, exploring fruit species all over the country and trying to improve them, cultivating new species in the botanical garden, inviting experts from the Soviet Union to China to perform research and give advice on cultivating fruit trees, carrying research on fruit trees and publishing monography on the taxonomy, etc.

Dejun Yu also stood firm to protect the science and resources of his own country. He told the history of plant exploration and plunder by western imperialists in China, and expressed his anger in an article. He mentioned that, plant hunters often collected seeds, specimens and even living economic and ornamental plants for their own countries. They even stole processing technology of plant products (such as tea making) and information of natural resources and environment, and sold plants or their seeds directly. Beautiful Chinese plants still living in European gardens, type specimens kept in their herbariums and Latin names of Chinese plants named after plant hunters are outstanding examples of their imperialism. Because of the imperial botany, China was greatly influenced on the development of plant resources and the progress of botanical sciences (Yu, 1952). Given this situation, Yu and other Chinese botanists tried their best to study specimens of Chinese plants during their visits to Europe, especially type ones.

Dejun Yu passed away on the afternoon of July 14, 1986. His last wish was that there would be no farewell ceremony for him, and his body would be donated to a hospital for pathological anatomy. His name was carved on a marble tombstone as the founder of Beijing BG. He will also be remembered in the history of botany and botanical gardens.
